# Supplementary material for: Optimized Xenograft Protocol for Chronic Lymphocytic Leukemia Results in High Engraftment Efficiency for All CLL Subgroups
Source: Int J Mol Sci. 2019 Dec 12;20(24):6277. doi: 10.3390/ijms20246277 (PMC6940872; doi:10.3390/ijms20246277)
Supplement: Supplementary file 1 [file ijms-20-06277-s001.zip › ijms-661483-supplementary/Decker Figure S1+2+3+4.pdf]

Figure S1

A

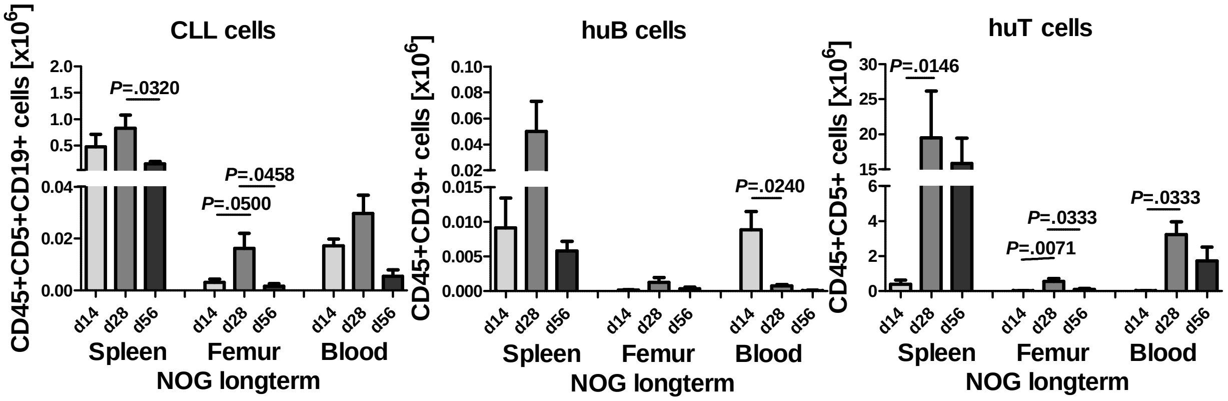

(A) Mean numbers of human CLL-, B- and T cells in the spleen, femur and PB of CLL-PBMC transplanted NOG mice at two, four and eight weeks after transplantation.

Figure S2

A

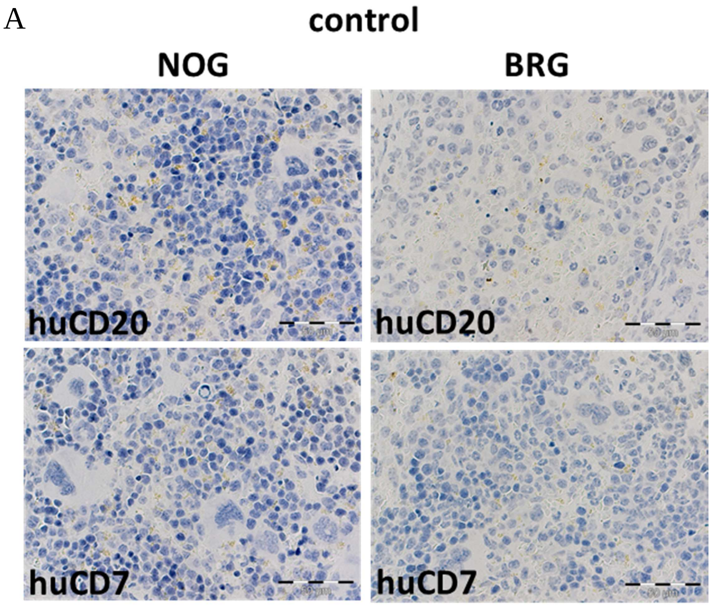

B

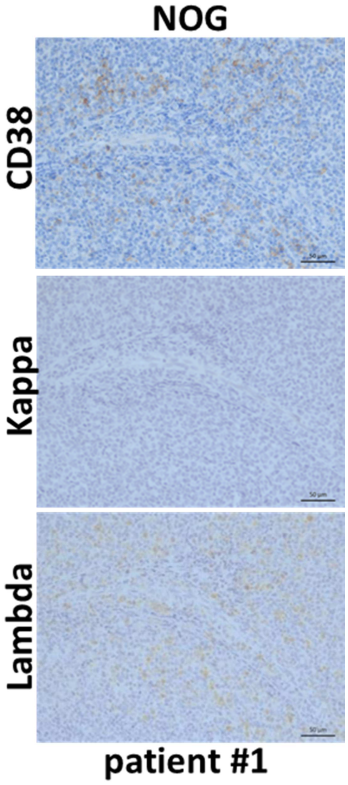

(A) IHC stainings of spleens of untransplanted NOG mice for human CD7 and CD20 at the age of 14 weeks. Image size: x400 original magnification for all images (Bar indicates 50µM). (B) IHC analysis of spleen samples from CLL-PBMC transplanted NOG mice stained for human CD38 and Kappa and Lambda light chain at day 28 after human cell injection (CLL patient #1). Image size: x200 original magnification for images (bar indicates 50µm).

Figure S3

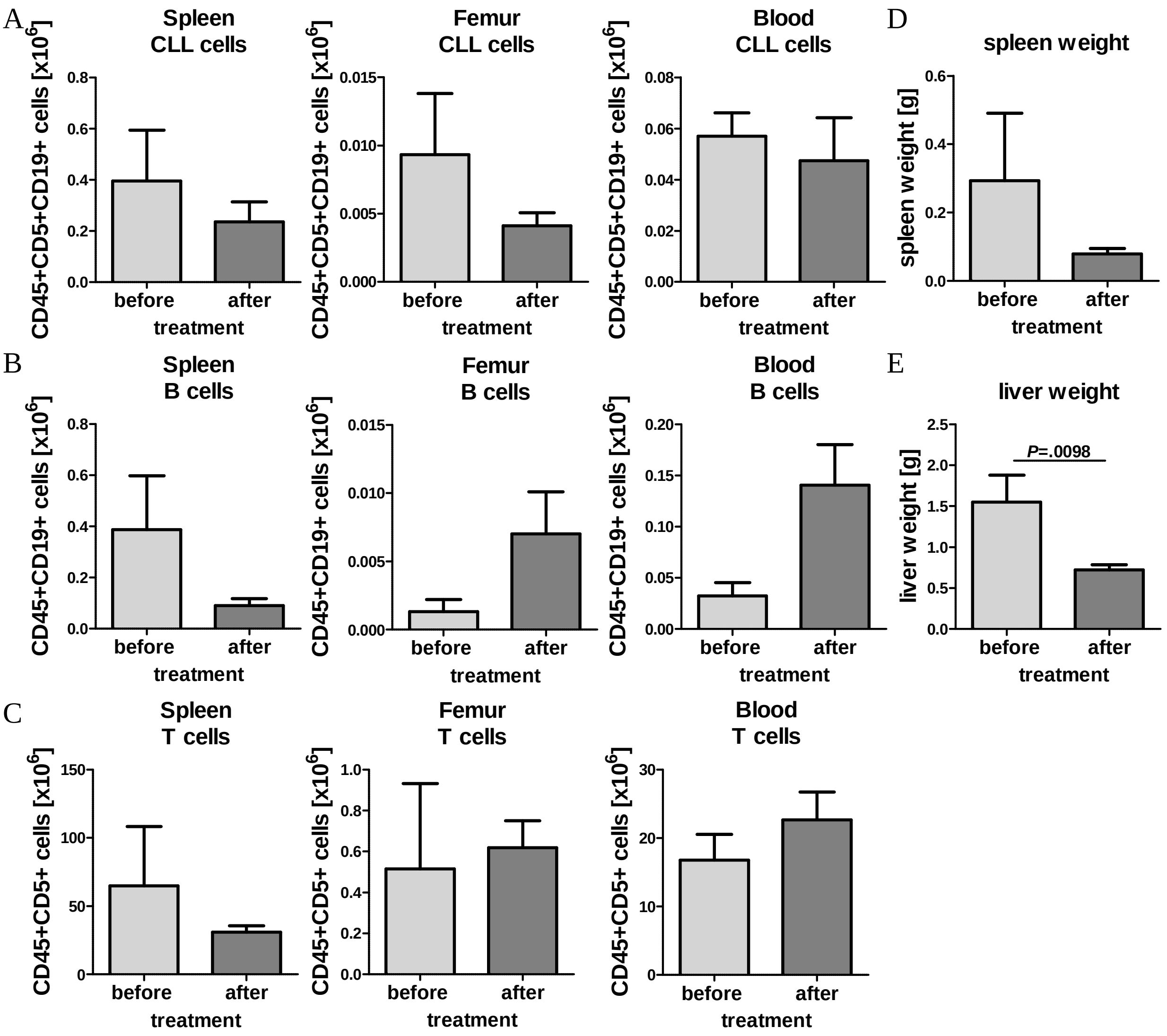

(A) Mean number of CLL cells in the spleen, femur and blood of NOG mice transplanted with CLL-PBMCs before and after chlorambucil treatment (CLL patient #8, n=3) four weeks after transplantation. (B) Mean number of human B cells in the spleen, femur and blood of NOG mice (CLL patient #8, n=3) transplanted with CLL-PBMCs before and after chlorambucil treatment 28 days after transplantation. (C) Mean number of human T cells in the spleen, femur and blood of transplanted NOG mice (CLL patient #8, n=3) four weeks after transplantation with CLL-PBMCs before and after chlorambucil treatment. (D) Mean spleen and liver weights of NOG mice transplanted with CLL-PBMCs before (CLL patient #8, n=3) and after chlorambucil treatment (CLL patient #8, n=3).

Figure S4

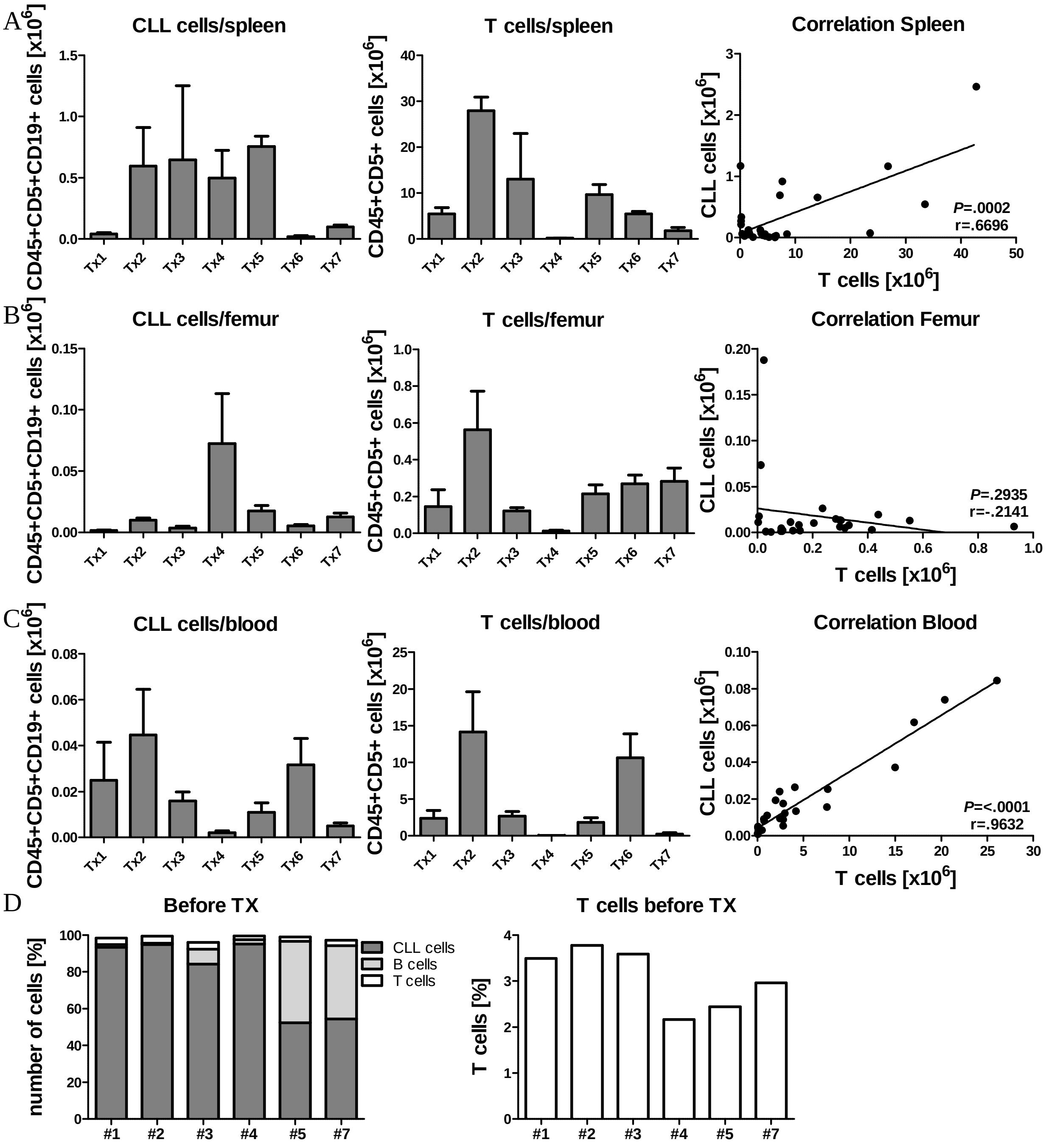

(A-C) Mean number of CLL- and T cells in the spleen (A), femur (B) and blood (C) of transplanted NOG mice (CLL patients #1-#7, n=4) four weeks after transplantation, and correlation of engrafted CLL and T cells in the spleen, femur and blood. (D) Distribution of CLL-, B- and T cells in the PB of CLL patients #1-#5+#7 before transplantation into NOG mice.
