# Supplementary material for: Optimized Xenograft Protocol for Chronic Lymphocytic Leukemia Results in High Engraftment Efficiency for All CLL Subgroups
Source: Int J Mol Sci. 2019 Dec 12;20(24):6277. doi: 10.3390/ijms20246277 (PMC6940872; doi:10.3390/ijms20246277)
Supplement: Supplementary file 1 [file ijms-20-06277-s001.zip › ijms-661483-supplementary/Decker Table S1_new.pdf]

Table S1

Patient characteristics of primary human CLL patients.

CLL	Sex	Age	IgVH	Genetic aberration	Rai stage	Previous therapy	Leukocytes [Tsd]	Monocytes [%]
#1	Ma	63	M	normal	2	None	124.1	0
#2	Ma	65	M	Trisomy 12	2	Chlorambucil	162.1	2
#3	Ma	70	M	Del 13q14	2	None	62.5	1
#4	Ma	74	M	normal	0	Chlorambucil	93.7	0
#5	Ma	79	M	Trisomy 12, Del 13q14	4	Chlorambucil	86.5	1
#6	Fe	71	M	normal	1	None	36.4	1
#7	Ma	58	M	Trisomy 12, Del 13q14	2	None	160.0	0
#8	Fe	36	M	Del 11q22	4	None/Chlorambucil	49.1	ND
#9	Ma	82	M	Del 13q14	1	Chlorambucil	81.0	0
#10	Ma	55	M	Del 13q14	2	None	102.4	1
#11	Ma	47	M	Del 13q14	0	None	71.3	ND
#12	Fe	44	UM	Del 13q14	1	None	76.7	3
#13	Ma	77	M	normal	0	Chlorambucil	114.9	ND
#14	Fe	74	UM	Del 11q22, Del 13q14	0	None	117.9	0
#15	Ma	72	ND	Del 13q14	ND	None	218.5	ND
#16	Ma	65	M	Trisomy 12	2	Chlorambucil	149.1	2
#17	Ma	79	UM	lossY	3	None	49.0	ND
#18	Ma	71	M	Del 13q14	2	None	57.0	1
#19	Fe	45	UM	Del 13q14	ND	None	86.8	3
#20	Fe	62	UM	Del 6q21, Del 6q23, Del 11q22, Del 13q14	1	None	128.1	ND
#21	Fe	67	M	Del 13q14	ND	None	62.0	ND
#22	Ma	78	M	Del 13q14	2	Chlorambucil	33.2	0
#23	Ma	59	M	Del 13q14	0	None	125.6	1
#24	Fe	45	UM	Del 11q, Del 13q14	2	FCR	9.2	0

Patient characteristics:

Ma=Male, Fe=Female, M=mutated, UM=unmutated, ND=not defined, None=no previous therapy, FCR=Fludarabine+Cyclophosphamide+Rituximab, Umbrella2=GS-4059+Entospletinib+Obinutuzumab
