# Supplementary material for: Optimized Xenograft Protocol for Chronic Lymphocytic Leukemia Results in High Engraftment Efficiency for All CLL Subgroups
Source: Int J Mol Sci. 2019 Dec 12;20(24):6277. doi: 10.3390/ijms20246277 (PMC6940872; doi:10.3390/ijms20246277)
Supplement: Supplementary file 1 [file ijms-20-06277-s001.zip › ijms-661483-supplementary/Decker Table S2.pdf]

Table S2

Engraftment of human cells

Engraftment of human CLL cells, B cells and T cells in NOG versus BRG mice. Each number represents the mean of 3-4 mice (*=3 mice) 28 days after transplantation.

**Spleen**	cells/spleen [mio]		human cells [%]		CLL cells [Tsd]		B cells [Tsd]		T cells [mio]	
CLL	NOG	BRG	NOG	BRG	NOG	BRG	NOG	BRG	NOG	BRG
#1	13.0	11.7	58.9	4.8	40.2	4.6	8.9	0.1	5.5	0.20
#2	90.4*	34.5	50.3	1.0	594.6*	16.9	296.6*	0.9	27.9*	0.40
#3	24.9	3.7	79.0	0.3	646.8	3.2	66.4	0.04	13.0	0.01
#4	47.3*	18.4	36.7	1.9	755.1*	4.4	630.7*	0.5	9.6*	0.04
#5	5.0	5.2	16.2	0.2	498.0	2.4	25.6	0.4	0.1	0.01
#6	22.0	6.5	26.3	42.1	18.7	9.3	8.5	3.9	5.4	2.40
#7	8.3	2.2	23.4	0.2	98.2	1.0	98.8	0.3	1.8	0.002

**BM**	cells/femur [mio]		human cells [%]		CLL cells [Tsd]		B cells [Tsd]		T cells [Tsd]	
CLL	NOG	BRG	NOG	BRG	NOG	BRG	NOG	BRG	NOG	BRG
#1	6.1	5.5	2.1	0.1	1.5	0.1	0.6	0.0	145.4	5.7
#2	8.5*	10.4	7.0	0.2	9.9*	0.2	8.9*	0.1	562.5*	1.9
#3	1.9	7.4	25.7	0.2	3.6	0.5	0.8	0.0	122.0	1.4
#4	5.7*	8.2	8.5	0.1	17.5*	0.3	19.7*	0.3	214.3*	5.6
#5	10.5	10.2	1.6	0.1	72.5	1.3	53.6	0.0	11.7	0.3
#6	3.7	8.2	8.2	0.6	5.3	1.5	0.6	0.3	269.7	44.7
#7	4.9	7.3	8.1	0.0	12.7	0.2	38.5	0.0	281.8	0.4

**PB**	WBC [$10^3/mm^3$]		human cells [%]		CLL cells [Tsd]		B cells [Tsd]		T cells [mio]	
CLL	NOG	BRG	NOG	BRG	NOG	BRG	NOG	BRG	NOG	BRG
#1	22.4	6.9	27.3	0.3	24.9	0.6	1.0	0.0	6.9	0.02
#2	24.4*	6.8	44.9	0.3	44.6*	0.3	43.7*	0.6	12.1*	0.01
#3	7.7	4.9	46.1	0.0	15.9	0.0	8.6	0.0	2.6	0.0
#4	2.7*	3.8	2.7	0.0	2.1*	0.5	0.1*	0.1	0.06*	0.02
#5	5.2	5.4	36.2	0.2	8.2	0.1	18.1	0.1	1.4	0.007
#6	20.2	4.5	49.8	9.4	31.6	0.6	0.6	0.1	10.7	0.42
#7	3.7	5.6	6.8	0.0	5.0	0.0	5.5	0.0	0.3	0.0
